# Rapid Detection of Measles Virus Using Reverse Transcriptase/Recombinase Polymerase Amplification Coupled with CRISPR/Cas12a and a Lateral Flow Detection: A Proof-of-Concept Study

**DOI:** 10.3390/diagnostics14050517

**Published:** 2024-02-29

**Authors:** Elena Pinchon, Steven Henry, Fanny Leon, Chantal Fournier-Wirth, Vincent Foulongne, Jean-François Cantaloube

**Affiliations:** Pathogénèse et Contrôle des Infections Chroniques et Emergentes, Etablissement Français du Sang, Université de Montpellier, Inserm, 34184 Montpellier, France; elena.pinchon@efs.sante.fr (E.P.);

**Keywords:** measles virus, reverse transcription, recombinase polymerase amplification, CRISPR/Cas12

## Abstract

The measles virus is highly contagious, and efforts to simplify its diagnosis are essential. A reverse transcriptase/recombinase polymerase amplification assay coupled with CRISPR/Cas12a and an immunochromatographic lateral flow detection (RT-RPA-CRISPR-LFD) was developed for the simple visual detection of measles virus. The assay was performed in less than 1 h at an optimal temperature of 42 °C. The detection limit of the assay was 31 copies of an RNA standard in the reaction tube. The diagnostic performances were evaluated on a panel of 27 measles virus RT-PCR-positive samples alongside 29 measles virus negative saliva samples. The sensitivity and specificity were 96% (95% CI, 81–99%) and 100% (95% CI, 88–100%), respectively, corresponding to an accuracy of 98% (95% CI, 94–100%; *p* < 0.0001). This method will open new perspectives in the development of the point-of-care testing diagnosis of measles.

## 1. Introduction

Measles is a highly contagious human infectious disease caused by a Morbillivirus of the *Paramyxoviridae* family [1,2,3]. This group contains animal viruses, including rinderpest virus (RPV), peste des petits ruminants virus (PPRV), cetacean morbillivirus, phocine distemper virus, canine distemper virus, and feline morbillivirus. This disease is characterized by general symptoms (fever, asthenia), oculo-respiratory signs (coryza, conjunctivitis), and a maculo-papular rash. Some life-threatening complications may occur, such as pneumonia and encephalitis, especially in infants under 1 year of age and adults over 20 years old, as well as immunocompromised people and pregnant women [4].

Despite the availability of an efficient vaccine, measle remains a global public health concern. The total number of measles virus infections increased by 300% in the first quarter of 2019, with a notable expansion in Europe [5]. Another recent report has described a global rise of 556% in the number of reported cases in 2019, with a 50% increase in global mortality since 2016. During 2021–2022, estimated measles cases increased by 18% and estimated measles deaths increased by 43% [6,7,8,9]. Currently, African countries (Nigeria, Liberia, Ethiopia) and Asian countries (India, Afghanistan) are the most affected (https://www.cdc.gov/globalhealth/measles/data/global-measles-outbreaks.html (accessed on 23 November 2022)).

In addition, measles vaccination campaigns have been postponed in 15 African countries due to the Covid pandemic, and 16.6 million children did not receive the measles vaccine during January 2020 to April 2021 [10]. In Africa, an alarming 400% increase in measles virus infections has been observed in the first quarter of 2022 compared with the same period the previous year (https://www.afro.who.int/news/vaccine-preventable-disease-outbreaks-rise-africa (accessed on 28 April 2022)), which amplifies the risk of larger outbreaks around the world. Furthermore, in a recent ECDC communicable diseases report, up to 595 new measles cases have been detected through epidemic surveillance in 10 European countries, including reports on new or ongoing outbreaks reported in Austria, Germany, France, and Romania (https://www.ecdc.europa.eu/communicable-disease-threats-report-week-41-2023 (accessed on 13 October 2023)). The measles virus genome consists of single-stranded (ss) negative-sense RNA and includes eight coding sequences for the following proteins: nucleocapsid (N), phosphoprotein (P), matrix (M), fusion (F), hemagglutinin (H), large protein (L), and two non-structural proteins V and C [2]. Measles virus is classified into eight genetic clades (A to H) based on full length N and H sequences. Routine genotyping is performed on partial sequences in the N region [11,12]. Measles virus has a high reproduction number (R0 = 12–18) [13,14], with a prolonged period of contagiousness extending from 3 to 5 days before the onset of the rash and persisting for up to 5 days afterwards [15].

Currently, the simplest approach available for the diagnosis of measles is based on peripheral blood serology [16]. However, direct testing for measles virus performed during the early phase of infection allows for the isolation of positive patients in order to prevent the spread of the disease. This detection would be useful (i) for people returning from epidemic areas; (ii) in subjects at risk of complications, such as infants, pregnant women, or immunocompromised patients; and (iii) in severe, complicated or atypical forms of measles. Nucleic acid testing is becoming the diagnostic test of choice, where resources allow their routine use. Conventional reverse transcription polymerase chain reaction (RT-PCR) and real-time RT-PCR have been developed as diagnostic tests for measles [12,17,18]. The combination of RT-PCR with IgM serology is recommended by the WHO. The amplicons are then genotyped in order to carry out the molecular epidemiology of the virus (https://www.technet-21.org/en/manual-introduction (accessed on 1 June 2018)). However, these approaches are time-consuming and require expensive equipment. Hence, new isothermal amplification methods avoiding complex devices have been elaborated. These tests can be combined with a wide range of real time detection methods including bioluminescence, fluorescence, turbidimetry or lateral flow migration [19,20]. An isothermal method, based on loop-mediated isothermal amplification (LAMP), has been described to detect the genome of the measles virus [21]. Nevertheless, this technique has several drawbacks associated with the need for a large number of primers with relatively complex designs and the inability to clone and sequence target products. A second isothermal method has also been described to detect measles virus and SARS-CoV-2 genomes. This method is based on recombinase polymerase amplification (RPA) coupled with a LAMP assay on a microfluidic-chip-based portable system [22]. RPA, which combines speed and simplicity, was first described in 2006 and has been successfully used for the detection of many pathogens such as bacteria, fungi, viruses, and parasites [23,24,25]. The RPA reaction requires a recombinase, ssDNA binding proteins and a polymerase. Recombinase separates a DNA target and catalyzes the hybridization of primers with the homologous sequences. The resulting structures are stabilized by ssDNA binding proteins and the polymerase initiates the primer extension reaction. The cyclic repetition at a temperature of 42 °C results in an exponential amplification of the target. RPA is a suitable method for field detection in a huge number of applications and this flexibility allows the use of various detection methods, such as fluorescence or lateral flow assays. RPA has been recently associated with the innovative clustered regularly interspaced short palindromic repeats (CRISPR)/Cas12a-targeted recognition system for viral detection [26,27,28]. CRISPR/Cas is an adaptative defensive system for bacteria against foreign genetic elements. It has recently been discovered that some Cas proteins, including Cas12a, have collateral ssDNA cleavage activity. The presence of a target-recognizing CRISPR/Cas12a RNA (crRNA) allows the cleavage of a non-target ssDNA reporter, which can then be visualized on a test strip. This property was exploited in a sensitive detection system named the DNA endonuclease-targeted CRISPR trans reporter (DETECTR) [26,29]. This approach was first described in two steps, which require the transfer of amplicons into the CRISPR/Cas12a reaction tube [26]. To avoid contamination, a one-tube format with the CRISPR/Cas12a mixture in the lid was developed [30,31].

Here we report the development and the performance evaluation of a one-tube RT-RPA CRISPR/Cas12a-based assay for the molecular detection of measles virus RNA. Our assay combines simultaneous reverse transcription and isothermal amplification using RT-RPA followed by CRISPR/Cas12a biosensing and a lateral flow detection (LFD). A lateral flow device is an immunochromatographic biosensor in which the results are visually obtained. The analytical sensitivity was studied on standard panels. Then, following evaluation in a proof-of-concept study of diagnostic performance on biological samples, we have demonstrated that our assay shows promise as a point-of-care test for Measles virus.

## 2. Material and Methods

### 2.1. Biological Materials

Amplirun^®^ Measles RNA control (genotype A, Vircell, Granada, Spain) titrated at 16,000 copies/µL was used as standard. The dilution occurred in molecular grade water (from 12,000 copies to 3.84 copies per reaction). Three external quality controls, the 2020, 2021, and 2022 Measles and Mumps External Quality Assessment (EQA) programs (QCMD, Glasgow, UK) were tested in this study (Supplemental Table S1). Core samples must be found correctly to approve the test sensitivity, whereas educational samples are used to challenge the test. Analytical specificity was evaluated on positive samples of various respiratory viruses from the QCMD panels, including severe acute respiratory syndrome coronavirus 2 (SARS-CoV-2, 1 sample), human parainfluenza virus Type 3 (PIV, 3 different samples), human metapneumovirus (hMPV, 1 sample), respiratory syncytial virus A (RSVA, 1 sample) and RSVB (3 different samples), human herpesvirus-6 (HHV-6, 3 different samples), varicella-zoster virus (VZV, 2 different samples), enterovirus (2 different samples), parvovirus B19 (2 different samples), and adenovirus (2 different samples). Diagnostic sensitivity was evaluated on a panel of 27 measles-positive RNA extracts from saliva. These samples were obtained from the University Hospital (CHU) of Montpellier, France. An input of 200 µL of samples was extracted with an Easy1 extractor (Qiagen, GmbH, Hilden, Germany) running the DSP Virus extraction kit (Qiagen) and a final 60 µL elution was used for real time RT-PCR during routine diagnostic test using the Bosphore Measles Detection Kit v1 (Anatolia Geneworks, Istanbul, Turkey). Left over RNA extracts were then tested with the RPA-CRISPR/Cas12 test. Diagnostic specificity was evaluated on a panel of 29 measles-negative saliva RNA extracts obtained from the CHU of Montpellier.

### 2.2. Genotyping

Measles samples were genotyped by sequencing the N-450 region, using primers that have been previously described [12]. Sequencing reactions were performed on the Applied GenAmp 2700 Peltier Thermal Cycler (Applied Biosystems, ThermoFisher, Waltham, MA, USA) using the ABI BigDye1 Terminator v3.1 Cycle Sequencing Kit (Applied Biosystems), following the manufacturer’s protocols. Sequencing was performed on the purified amplicon template (Qia-quick PCR purification kit, Qiagen) using both forward and reverse primers. The fluorescent-labeled fragments were purified from the unincorporated terminators with the BigDye XTerminator1 Purification Kit (Applied Biosystems). The samples were injected for electrophoresis in an ABI 3500 Dx Genetic Analyzer (Applied Biosystems).

### 2.3. Design of Measles Specific Primers, Exo Probe and crRNAs

Based on RPA primers directed against PPRV [32], homologous primer sequences (Meas_NS1, Meas_NR1) targeting the N region of the measles virus genome were designed. Sequences were retrieved from GenBank and aligned using Clustal W software version 2.0. The complementarity of the RPA primer sequences was confirmed with sequences belonging to the different genotypes of the measles virus (Supplemental Figure S2). An exo probe (Meas_Probe) used for RT-RPA was designed according to RPA guidelines (TwistDx, Cambridge, UK).

Two guides (crRNA1 for genotypes A and D8 and crRNA2 for genotype B3) were designed according to Cas12a properties (protospacer adjacent motif (PAM): TTTV). All the oligonucleotides (primers, exo probes, crRNA and reporter ssDNA, see Table 1) were synthesized and provided by Kaneka Eurogentec (Seraing, Belgium). 

### 2.4. Real-Time RT-RPA Reaction Conditions

After hybridization of the probe with the template, the real-time detection was based on the cleavage of tetrahydrofuran (THF) between the fluorophore and the quencher thanks to *E. coli* exonuclease III. The real-time RPA assay was performed in a 50 µL volume using the TwistAmp^®^ Liquid Exo kit (TwistDx, Cambridge, UK). The reaction mixture included 25 µL of 2× reaction buffer, 2 µL of forward Meas_NS1 primer (10 µM), 2 µL of reverse Meas-NR1 primer (10 µM), 0.6 µL of Meas_Probe (10 µM), 1 µL of ROX 50× (Thermo Fischer Scientific, Illkirch, France), 3.4 µL of dNTPs (25 mM, Thermo Fischer Scientific), 5 µL of probe E mix (TwistDx), 1 µL of SuperScript II (200 U/µL Thermo Fischer Scientific), 2.5 µL of core reaction (TwistDx), and 1 µL of Exonuclease III (TwistDx). Extracted RNA (4 µL) was added to each tube. The addition of 2.5 µL of magnesium acetate (280 mM) initiated the RPA reaction. After 4 min of incubation at 42 °C in a Biometra TAdvanced (Analytik Jena GmbH, Jena, Germany), the reaction was carried out in a Step One Plus Applied Biosystem device (Thermo Fischer Scientific) at a temperature of 42 °C. Real-time detection was performed by quantifying the fluorescent signal ratio (FAM [6-carboxyfluorescein]/ROX [carboxyrhodamine]) every minute. The ROX passive reference fluorochrome was added to the reaction to normalize the well-to-well signal differences.

### 2.5. RT-RPA CRISPR/Cas12a Reaction and Lateral Flow Detection

The RT-RPA CRISPR/Cas12a reaction was performed as described [30] with minor modifications. The RPA pellet (TwistAmp basic RPA kit, TwistDx) was resuspended with 29.5 μL of rehydration buffer. The 25 µL of reaction mix included 14.75 μL of rehydrated buffer, 0.8 μL of forward primer (10 µM), 0.8 μL of reverse primer (10 µM), 0.24 µL of SuperScript IV (200 U/µL, Thermo Fischer Scientific), 0.48 µL of RNase H (5000 U/mL, New England Biolabs, Evry, France), 1.68 μL of nuclease-free water, 1.25 μL of magnesium acetate (280 mM) and 5 μL of extracted RNA template.

The CRISPR/Cas12a reaction mix consisted of 4.5 μL 10× of NEB Buffer 2.1 (New England Biolabs), 2 μL of Cas12a (1 µM) (New England Biolabs), 2 μL of crRNA (300 nM), 5 μL of ssDNA reporter (1 µM), and 6.5 μL of nuclease-free water. This 20 μL solution of CRISPR/Cas12a mix was added to the lid of the centrifuge tube before closing.

**Figure 1:**
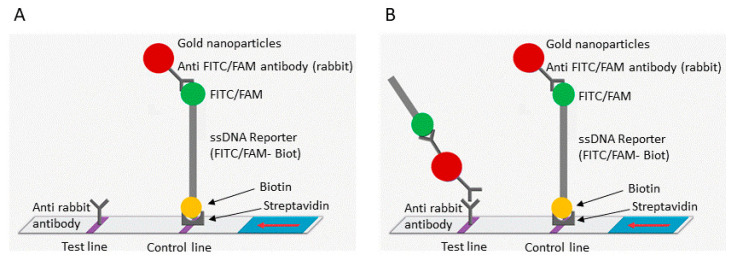
Schematic representation of the lateral flow detection test. (**A**) Uncleaved reporter molecules are captured at the first detection line (control line) and indicate a negative result. (**B**) Cas12 cleavage activity generates a supplementary signal at the second detection line (test line) and indicates a positive result. The red arrow indicates the direction of the flow.

The tube was placed in a thermoblock at 42 °C and incubated for 30 min. The CRISPR/Cas12a mixture added in the lid was then mixed with the RPA reaction via a short pulse and incubated for a further 20 min at 42 °C. The amplicons (15 µL) were loaded on an immunochromatographic strip (HybriDetect, Milenia Biotech, Gießen, Germany), which was then immersed for 5 min in a tube containing 90 µL of migration buffer at room temperature. The results were visualized directly by the naked eye (Supplemental Figure S1, Figure 1).

### 2.6. Statistical Analysis 

Accuracies and 95% CIs for a proportion were calculated using GraphPad software version 10.0.3 (GraphPad Prism, San Diego, CA, USA).

## 3. Results

### 3.1. RPA Primer Pair Validation

**Figure 2:**
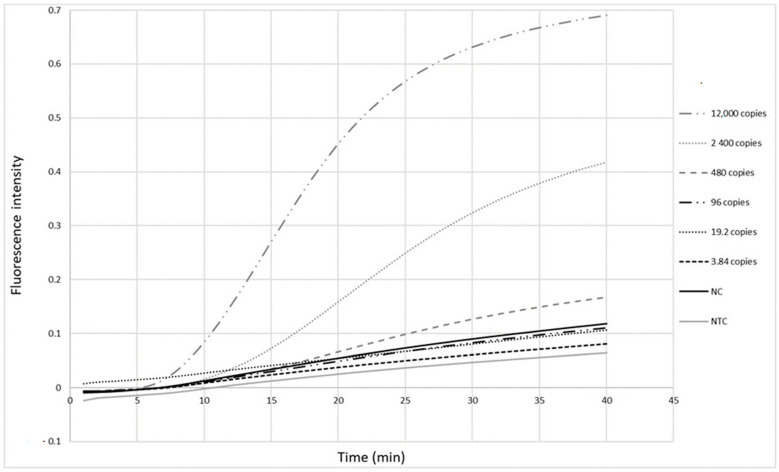
Detection limit of the measles RPA-real time assay. Five-fold serial dilutions of Vircell measles RNA control ranging from viral loads of 12,000 to 3.84 copies in the reaction tube were tested. Each dilution was tested twice. Cut-off value is 1.51 (mean value of negative samples + 3 standard deviation). NC: negative control, NTC: no template control.

The primers (Table 1) were first tested with the RPA exo assay on a panel of five-fold serial dilutions (12,000 to 3.84 copies per reaction) of a standard measles virus RNA sample from Vircell. Fluorescence signals were observed as soon as 10 min after the reaction was initiated (Figure 2). The detection limit of this real-time RPA was 480 copies in the reaction tube, corresponding to 120 copies/µL RNA.

### 3.2. Analytical Performances of RT-RPA-CRISPR-LFD Assay on Reference Standard

**Figure 3:**
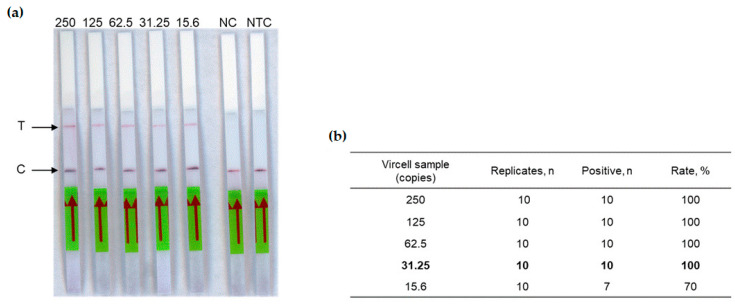
Analytical sensitivity of the measles RT-RPA-CRISPR-LFD assay. (**a**) Two-fold serial dilutions of the Vircell standard control ranging from viral loads 250 to 15.6 copies in the reaction tube were tested. T: Test line. C: Control line. NC: Negative control (negative plasma was used as template), NTC: No template control. The red arrow indicates the direction of the flow. (**b**) Limit of Vircell control standard (in bold), calculated on ten replicates of each sample.

The next step of this study was to test the complete RT-RPA-CRISPR-LFD workflow. The detection limit of our assay was determined using the crRNA1 (Table 1) on two-fold dilution range (from 250 to 15.6 copies in the reaction tube) of the Vircell standard measles virus sample. Each dilution was tested ten times, allowing a detection limit of 31.25 copies in the reaction tube (Figure 3), corresponding to 6.25 copies/µL RNA.

Analytical specificity was evaluated on a panel of 20 respiratory viruses or viruses involved in febrile rashes (see the list in the method section) and did not result in nonspecific reactions (Table 2).

### 3.3. Diagnostic Performances of RT-RPA-CRISPR-LFD Assay on Biological Samples

To analyze the sensitivity and the specificity of the assay, the 2020, 2021, and 2022 Measles and Mumps External Quality Assessment QCMD were first tested blindly (Supplemental Table S1). Each panel contained (i) positive measles core samples, (ii) negative core samples, (iii) educational measles samples and (iv) educational or core mumps samples. The measles core samples must be found to approve the test sensitivity whereas the educational samples were allowed to challenge and were not required to validate the test. All positive measles core samples (*n* = 11) were correctly detected (Z score = 0) without false negative results. No false positive results were obtained on negative core or mumps core samples.

**Figure 4:**
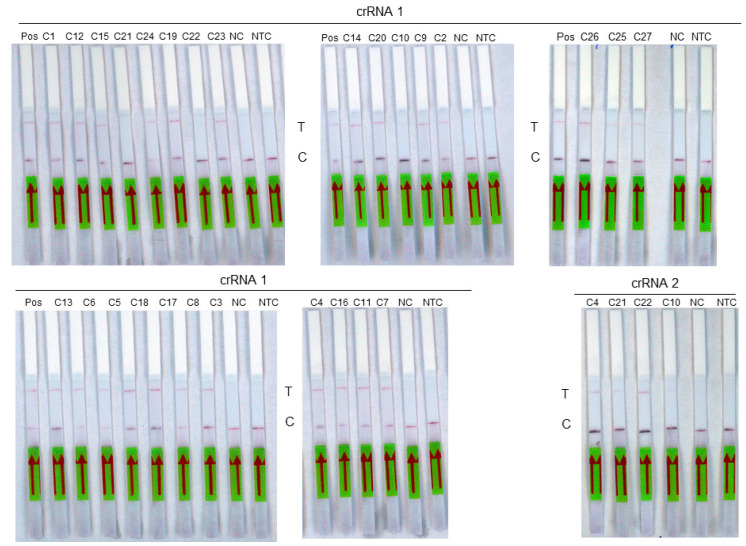
Analysis of a panel of 27 measles virus samples using RT-RPA-LFD assay. Each sample is identified on the top of its strip. Pos: Positive sample, T: test line. C: control line. NC: negative control, NTC: non-template control. The red arrow indicates the direction of the flow.

The diagnostic sensitivity was then evaluated on a panel of 27 RNA extracts from samples previously tested positive by the routine molecular method (Figure 4). These include 17 samples of genotype D8, 1 sample of genotype A, 3 samples of genotype B3, and 6 samples that could not be genotyped (Table 3). The diagnostic specificity was evaluated on a panel of 29 measles-negative saliva RNA extracts used as negative controls. Using the crRNA 1 guide, the 18 measles samples of genotypes A and D8 as well as the 6 non genotyped samples were detected by RT-RPA-Crispr-LFD assay. Among the three genotype B3 samples tested with the crRNA2 guide, two were detected. A sensitivity of 96% (95% CI, 81–99%) was obtained for the assay. A specificity of 100% (95% CI, 88–100%) was observed. The accuracy of the test was 98% (95% CI, 94–100%; *p* < 0.0001).

## 4. Discussion

In the context of measles outbreaks, a rapid virologic diagnostic is of great interest for appropriate medical care and control of spread. The currently recommended method for the routine diagnosis of acute measles virus infection involves IgM testing or direct antigen detection. Recently, a lateral flow rapid diagnostic test detecting measles-virus-specific IgM has been described and evaluated [33,34]. However, nucleic acid testing may be considered useful in the event of inconclusive results or for people returning from epidemic areas and persons at risk. Measles RNA detection may be particularly relevant in the very early stage of the disease, likely before the rise of IgM. Furthermore, beyond this individual benefit, an early strategy based on direct viral detection is an efficient tool for controlling contagion and spread of an infection. A rapid test for the detection of the measles virus genome should ideally be combined with a rapid test for the detection of measles-virus-specific IgM.

The aim of this work was to develop a fast and flexible molecular method, without the need for automated devices such as thermocyclers, that allows for the simple detection of measles virus from biological fluids. We combined reverse transcriptase, isothermal amplification by RPA, CRISPR/Cas12a biosensing and an immunochromatographic strip associated with naked-eye visualization. To limit potential aerosol contamination, the RT-RPA and CRISPR/Cas12a steps were carried out in the same reaction tube. 

The analytical sensitivity of the RT-RPA-CRISPR-LFD assay was 6.25 copies/µL RNA, whereas the sensitivity of the RT-RPA exo fluorescence assay was 120 copies/µL RNA. The CRISPR/Cas12a thus allowed for a substantial gain in sensitivity of about 19 fold. A comparable sensitivity, close to 8.8 copies/µL RNA, has been previously described in the isothermal assay that associated RT-LAMP and lateral flow detection [21]. However, our RT-RPA-CRISPR assay presents the advantages of being easier to design, rapid and conducted at a single and low temperature of 42 °C. Unless the use of the crRNA improves analytical sensitivity, the presence of mismatches in the sequence targeted by the crRNA may result in false negative results. 

In this proof-of-concept study, the performance evaluation was mainly conducted on D8 genotype samples corresponding to the recently circulating measles virus genotype in Europe during the latest outbreaks. The strains of this genotype were correctly detected, making it possible to validate the crRNA1 guide. All measles virus genotypes could not be tested here as corresponding samples were not available in the laboratory. Only two strains of genotype B3 out of 3 were detected using the crRNA2. The low number of strains belonging to this genotype did not allow validation of this second guide. 

However, considering the flexibility of both primers and crRNA design, the assay could be rapidly adapted to detect any circulating measles virus genotype. Alongside its high sensitivity, our RT-RPA-CRISPR-LFD assay also showed high specificity, as no false positive was identified on any other respiratory viruses tested. It also meets most of the ASSURED criteria for near point-of-care tests. Our assay is affordable (around USD 4), specific, sensitive, and easy to use, with minimal steps. This system operates at 42 °C in a simple thermoblock and at room temperature for the immunochromatographic visualization. It does not require specific and costly equipment, such as thermocyclers, and is easily deliverable in the field. An assay that fills these requirements for near-point-of-care testing is of particular interest for measles management considering the high reproduction rate of the measles virus and the need for rapid responses to control the spread of the virus, especially in low-income countries. 

The procedure we have described needs further improvement before it can be ready for field deployment. If performances of our biosensing system are promising, one main issue remaining for routine development could be the simplification of sample pretreatment. Many previous studies have shown that these biosensing processes based on CRISPR-Cas are robust and tolerant, allowing the use of cruder sample inputs rather than previously extracted RNA. Even if the counterpart will probably incur a slight drop in sensitivity, the latter must be assessed in balance with the benefit of speed and ease of use for a point-of-care system.

In conclusion, the preliminary results obtained using this flexible RT-RPA-CRISPR-LFD assay with a naked-eye visualization opens the way for improving rapid and simple strategies, supporting routine measles surveillance and control.

## Figures and Tables

**Table 1:** Sequences of oligonucleotides.

Oligo Name	Sequence	Position
Meas_NS1	CAA-AGG-CGG-TTA-CGG-CYC-CAG-ACA-CRG-CAG-CTG-AT	643-677
Meas_NR1	ACC-ACA-TCC-AAC-CAT-TTT-CTC-TCC-AAT-CTA-AAT-TC	763-729
Meas_Probe	AAT-CTA-AAT-TCA-CCA-ACT-ACC-CTT-CTT-TGT-(dT-FAM)G(THF)-G(dT-BHQ)G-TAY-TTT-ATC-CAC-C-(C3)	735-691
crRNA1	UAA-UUU-CUA-CUA-AGU-GUA-GAU-UUG-GGU-GUA-CUU-UAU-CCA-CCU-UC	710-688
crRNA2	UAA-UUU-CUA-CUA-AGU-GUA-GAU-UUG-GGU-GUA-UUU-GAU-CCA-CCU-UC	710-688
Reporter	6-FAM-TTATT-Biotin	

Probe modifications: FAM: 6-carboxyfuorescein; THF: tetrahydrofuran; BHQ: black hole quencher; spacer-C3: 3′ phosphate blocker. Nucleotide position according to GenBank access number NC_001498.

**Table 2:** Performances of the measles RT-RPA-CRISPR-LFD assay.

Sample Type	Samples Correctly Detected	Analytical Specificity ^†^ %	Diagnostic Sensitivity * % (95% CI)	Diagnostic Specificity ^§^ %	Accuracy ^‡^ % (95% CI)
Measles (crRNA1)	24/27		89 (71–96)		94 (87–100)
Measles (crRNA1 and 2)	26/27		96 (82–99)		98 (93–100)
Healthy	29/29			100	
Other viruses ^§^	20/20	100			

Detailed results can be found in Table 3 and Figure 4. ^§^ SARS-CoV-2, PIV3 MPV, RSV A and RSV B, HHV-6, VZV, enterovirus, parvovirus B19, adenovirus. CI: Confidence interval. ^†^ [number of negative samples/(number of negative samples + number of false-positive samples)] × 100. * [number of positive samples/(number of positive samples + number of false-negative samples)] × 100. **^§^** [number of negative samples/(number of negative samples + number of false-positive samples)] × 100. ^‡^ [(number of negative samples + number of positive samples)/(number of negative samples + number of positive samples + number of false-negative samples + number of false-positive samples)] × 100.

**Table 3:** Detection of measles virus RNA using four detection methods: RT-PCR, visualization of amplicon on an agarose gel, by RT-RPA exo fluorescence assay, and by RT-RPA-CRISPR-LFD (see Section 2).

RNA Extract	Genotype	RT-PCR	RT-RPA			
		Fluorescence	Agarose	Fluorescence	crRNA1	crRNA2
		(Ct)	Electrophoresis	(TwistAmp Exo)		
C1	D8	19.8	+	+	+	
C2	D8	19.9	+	+	+	
C3	ND	20.1	+	+	+	
C4	D8	20.5	+	+	+	
C5	D8	20.7	+	+	+	
C6	D8	21.1	+	+	+	
C7	D8	21.9	+	+	+	
C8	D8	22.1	+	+	+	
C9	D8	23.6	+	+	+	
C10	B3	23.8	+	+	−	−
C11	D8	23.8	+	+	+	
C12	D8	24.5	+	+	+	
C13	D8	25	+	+	+	
C14	D8	26	+	+	+	
C15	D8	26.1	+	+	+	
C16	ND	26.2	+	+	+	
C17	D8	26.5	+	+	+	
C18	D8	26.8	+	+	+	
C19	D8	27	+	+	+	
C20	A	27.2	+	+	+	
C21	B3	28.5	+	+	−	+
C22	B3	28.9	+	+	−	+
C23	D8	29.5	+	+	+	
C24	ND	31	−	+	+	
C25	ND	31	−	−	+	
C26	ND	35.5	−	−	+	
C27	ND	36.1	−	−	+	

## Data Availability

The data presented in this study are available on request from the corresponding author. The data are not publicly available due to privacy.

## Supplementary Materials

The following supporting information can be downloaded at: https://www.mdpi.com/article/10.3390/diagnostics14050517/s1, Figure S1: Workflow. Figure S2: Nucleotide sequence alignment of different measles virus genotypes (A to H2); Table S1: Detection of measles virus from the QCMD.
