# Supplementary material for: Rapid Detection of Measles Virus Using Reverse Transcriptase/Recombinase Polymerase Amplification Coupled with CRISPR/Cas12a and a Lateral Flow Detection: A Proof-of-Concept Study
Source: Diagnostics (Basel). 2024 Feb 29;14(5):517. doi: 10.3390/diagnostics14050517 (PMC10930384; doi:10.3390/diagnostics14050517)
Supplement: Supplementary file 1 [file diagnostics-14-00517-s001.zip › diagnostics-2672445-supplementary.pdf]

Supplemental Figure S1. Workflow

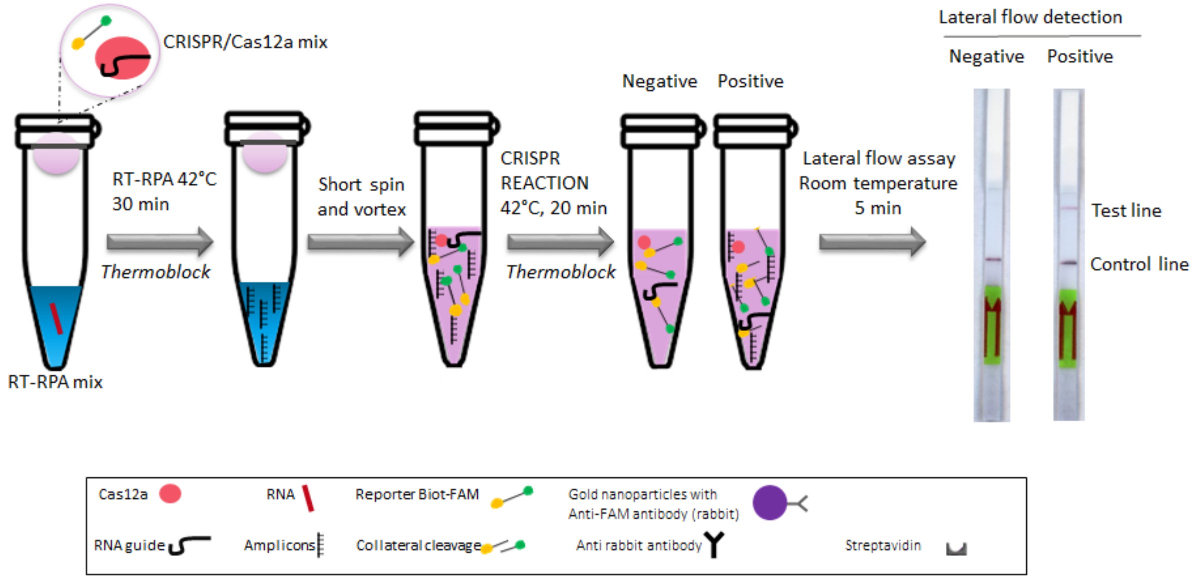

Supplemental Figure S2. Nucleotide sequence alignment of different measles virus genotypes (A to H2).

```
U01987_A        CAAAGGCGGT TACGGCCCCA GACACGGCAG CTGATTCGGA GCTAAGAAGG TGGATAAAGT ACACCCAACA
U01994_B2       .......... .......... .......... .......... .......... ........A. ..........
L46753_B3       .......... .......... .....A.... .......... .......... ........A. ..........
M89921_C2       .......... .......... .......... .......... .......... .......... ..........
DQ839360_D2     .......... ......T... .......... .......... .......... ........A. ..........
U01977_D3       .......... .......... .......... .......... .......... .......... ..........
U01976_D4       .......... .......... .......... .......... .......... .......... ..........
L46758_D5       .......... .......... .......... .......... .......... .......... ..........
L46750_D6       .......... .......... .......... .......... .......... .......... ..........
AF504047_D7     .......... .......... .......... .......... .......... .......... ..........
AF280803_D8     .......... .......... .......... .......... .......... .......... ..........
U01974_G1       .......... .......... .......... .......... .......... .......... ..........
AF171232_G2     .......... .......... .......... .......... .......... ........A. ..........
AF045212_H1     .......... ...T...... .......... .......... .......... .......... ..........
AF045217_H2     .......... .......... .......... .......... ...T...... .......... ..........

U01987_A        AAGAAGGGTA GTTGGTGAAT TTAGATTGGA GAGAAAATGG TTGGATGTGG T
U01994_B2       ......A... .......... .......... .......... .......... .
L46753_B3       ......A... .......... .......... .......... ........T. .
M89921_C2       .......... .......... .......... .......... .......... .
DQ839360_D2     .......... .......... ..C....... .......... .......... .
U01977_D3       .......... .......... .......... .......... .......... .
U01976_D4       .......... .......... .......... .......... .......... .
L46758_D5       .......... .......... .......... .......... .......... .
L46750_D6       .........T .......... .......... A......... .......... .
AF504047_D7     .......... .......... .C........ .......... .......... .
AF280803_D8     .......... .......... .......... .......... .......... .
U01974_G1       .......... .......... .......... .......... .......... .
AF171232_G2     .......... ..C....... .C........ .......... ........A. .
AF045212_H1     .......... ..C....... .......... .......... .......... .
AF045217_H2     .......... .......... .......... .......... .......... .
```

Positions of primers are framed. Position of crRNAs is undelined

	Sample	RT-RPA-CRISPR-LFD assay
QCMD 2020	Negative Core	-
	Measles Core	+
	Measles Core	+
	Mumps Educational	-
	Measles Educational	-
	Negative Core	-
	Mumps Educational	-
	Measles Educational	-
	Mumps Educational	-
	Measles Core	+
QCMD 2021	Measles Core	+
	Mumps Educational	-
	Measles Core	+
	Measles Educational	-
	Negative Core	-
	Measles Core	+
	Mumps Educational	-
	Mumps Educational	-
	Measles Core	+
	Mumps Educational	-
QCMD 2022	Measles Core	+
	Mumps Core	-
	Measles Core	+
	Mumps Core	-
	Measles Core	+
	Mumps Core	-
	Measles Core	+
	Mumps Educational	-
	Measles Educational	-
	Negative Core	-

Supplemental Table S1. Detection of measles virus from the QCMD (Quality Control of Molecular Diagnostic)
